# Enzymatic Polymerization of Cyclic Monomers in Ionic Liquids as a Prospective Synthesis Method for Polyesters Used in Drug Delivery Systems

**DOI:** 10.3390/molecules20010001

**Published:** 2014-12-23

**Authors:** Urszula Piotrowska, Marcin Sobczak

**Affiliations:** 1 Department of Inorganic and Analytical Chemistry, Faculty of Pharmacy, Medical University of Warsaw, Banacha 1, Warsaw 02-097, Poland; E-Mail: marcin.sobczak@wp.pl; 2 Chair of Chemistry, Department of Organic Chemistry, Faculty of Materials Science and Design, Kazimierz Pulaski University of Technology and Humanities in Radom, Chrobrego 27, Radom 26-600, Poland

**Keywords:** biomedical polymers, macromolecular conjugates of drugs, enzymatic ring opening polymerization, ionic liquids, biodegradable polyesters, drug delivery systems

## Abstract

Biodegradable or bioresorbable polymers are commonly used in various pharmaceutical fields (e.g., as drug delivery systems, therapeutic systems or macromolecular drug conjugates). Polyesters are an important class of polymers widely utilized in pharmacy due to their biodegradability and biocompatibility features. In recent years, there has been increased interest in enzyme-catalyzed ring-opening polymerization (e-ROP) of cyclic esters as an alternative method of preparation of biodegradable or bioresorbable polymers. Ionic liquids (ILs) have been presented as green solvents in enzymatic ring-opening polymerization. The activity, stability, selectivity of enzymes in ILs and the ability to catalyze polyester synthesis under these conditions are discussed. Overall, the review demonstrates that e-ROP of lactones or lactides could be an effective method for the synthesis of useful biomedical polymers.

## 1. Introduction

A large part of currently used chemotherapeutic agents are low molecular weight compounds (e.g., nucleic acids, peptides, enzymes). They are characterized by fast metabolism and fast excretion from the organism as well as adverse biodistribution and low therapeutic selectivity. To solve this problem, conventional therapies focus on dosage of active pharmaceutical ingredients (APIs) in a high frequency manner. The disadvantages of such a solution are the possibility of adverse reactions resulting in variable concentration of chemotherapeutic agents or their excessive level in a healthy tissues [1].

A promising approach to safely achieving desired therapeutic effect of APIs are polymer-based drug delivery systems (DDS) [2]. Polymeric carriers exhibit suitable pharmacokinetics, biodistribution, and pharmacological efficacy. They increase the bioavailability of the therapeutic agents by modification of the API’s solubility. Polymeric carriers also provide protection against endocytosis, phagocytosis, enzymatic degradation and cause reduction in antigenic activity of the drug leading to less pronounced immunological body responses. The tissue types or disease-specific structures are taken into account. It results in appropriate matching of distribution place to the drug release mechanism (active or passive transport) [3].

A few fundamental requirements should be met for any polymer material to be used as a drug carrier: excellent biocompatibility, precision biodistribution and predictable release profile of the therapeutic substances. Rationally designed polymeric carriers have some advantages, e.g., elimination of multiple therapeutic substances which are used during the day or reducing the daily dose. Therefore they provide required toxicological safety while maintaining a high efficiency [4,5]. Development of a new DDS is highly prioritized direction of the modern pharmacy and medicine.

Aliphatic polyesters and copolymers of cyclic esters and carbonates are representatives of a family of polymers commonly used in DDS technology. In this paper, we aim to demonstrate some of the current directions in developing synthesis methods for biodegradable or bioresorbable polyester carriers.

## 2. Polymeric Carriers

There are currently two main methods for obtaining polymer-based DDS. The first method consists of the usage of therapeutic agents immobilized *via* incorporation into a variety of polymeric biomaterials. The active substances are suspended or dissolved into the polymer matrix. The API’s release rate depends on the hydrophilic-hydrophobic properties, degree of crystallinity, molecular weight, polydispersity and architecture of the polymer carriers, as well as the therapeutic agent’s properties (e.g., solubility, concentration gradient) and process conditions (pH, temperature, ionic strength, presence of enzymes, *etc*.). It is also possible to modify drug release kinetics by optimizing the matrices’ synthesis parameters. The release of APIs from biodegradable matrices can be governed by several mechanisms: (1) diffusion of therapeutic agents through the polymer matrix; (2) degradation (erosion) of the polymer; (3) by the influence the osmotic pressure. However, many biodegradable DDS are very complex and the release occurs through a combination of several concurrent mechanisms [6,7].

## 3. Macromolecular Conjugates of Active Substances

Polymeric prodrugs (or macromolecular conjugates of drugs) are a specific type of therapeutic agents in which APIs are linked to a macromolecular carrier (promoiety) through a physiologically labile bond [8,9]. Release of small therapeutic molecules from those systems can be achieved by polymer hydrolysis. APIs are subsequently metabolized into the active precursors through enzymatic or non-enzymatic process*.* The process takes place prior to their (the APIs’) absorption, after absorption, or at a specific site of the system. Macromolecular prodrugs are very promising API carriers. The high potential of these hybrid materials stems from the synergistic interaction between the elements of their composition [10,11].

**Figure 1:**
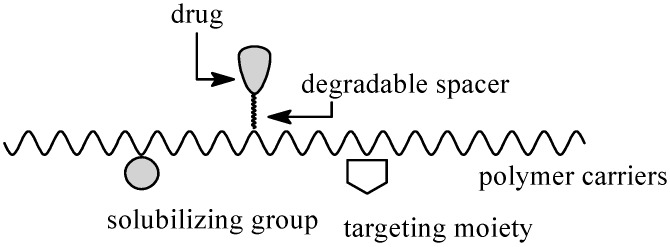
Polymer-drug conjugate model proposed by Ringsdorf.

In 1975 Helmut Ringsdorf proposed a model for the rational design of polymeric prodrug (Figure 1) [12,13]. This model is based on a polymeric matrix linking therapeutic substances *via* covalent bonds. Several types of labile bonds (e.g., amide, carbonate, ester, urethane) can be used to form biodegradable or bioresorbable polymeric-APIs conjugates.

The therapeutic molecules can be incorporated into the polymer chain, may be linked at the end of macromolecular chains or can form pendant groups on the polymeric chain. Prodrugs usually include several components: (1) polymeric carrier; (2) API; (3) targeting moiety *i.e.*, antibodies, sugars, peptides, proteins or oligonucleotides, which are responsible for the selectively deliver of the APIs to the appropriate tissue; (4) solubilizing group, which is responsible for an increased solubility of low solubility or insoluble therapeutic agents [9,14,15,16].

The use of a properly designed carrier for targeted pharmaceutical delivery leads to an increased accumulation of therapeutic molecules in the target tissue. This is the result of: (1) the appropriate molecular weight of the polymer (a slow degradation of the macromolecules extends the biological half-life of the drug in the tissue); (2) structure and origin of the carrier (synthetic or natural polymers such as proteins, polysaccharides); (3) presence of a specific targeting moiety [11,17].

Polymeric carriers are able to provide active or passive targeting of the APIs specifically to the site of their action. Passive targeting usually depends upon an enhanced permeation and retention (EPR) effect and is widely used as an approach for targeting tumor cells due to the specific differentiated properties of tumor tissues (enhanced vascular permeability, poor lymphatic drainage) [18,19]. High concentrations of pharmaceuticals in tumors have been achieved by entrapping and accumulating API molecules for prolonged periods. Therapeutic agents may also be transported into the cell by concentration differences of APIs on the opposite sides of a cellular membrane [20].

## 4. Aliphatic Polyesters as a Carriers of Therapeutic Agents

Biopolymers and their derivatives are commonly used in pharmacy as biomaterials for DDS. Polymers derived from synthetic monomers also show excellent delivery properties. The most promising group are biocompatible and biodegradable polymers (Table 1). They can be readily hydrolyzed into non-toxic and removable products and then excluded by normal metabolic processes [7,15,21].

Bioresorbable and biodegradable aliphatic polyesters have been extensively investigated as matrices for DDS. They are commonly known as synthetic polymers for biomedical application. The presence of suitable functional groups (hydroxyl and carboxyl) in their molecules allows covalent coupling of therapeutic agents while maintaining their activity. These polyesters include lactides (e.g., dimer of lactic acid, glycolide), lactones (β-propiolactone, δ-valerolactone, ε-caprolactone) and copolymers of various heterocyclic monomers such as *rac*-lactide, l-lactide, glycolide, ε-caprolactone and trimethylene carbonate. They are degraded *in vivo* by hydrolysis reactions into non-toxic products such as glycolic acid, lactic acid or other compounds that become involved in the tricarboxylic acid cycle and are subsequently excreted as carbon dioxide and water. The kinetics of polyester degradation can be tailored by the modifying their morphologies and hydrophilicities during the synthesis [15,21,22].

**Table 1:** Examples of DDS obtained from polyesters.

Polyesters	Therapeutic Agent	Ref.
poly(lactide-*co*-glycolide) (PLG)	Amphotericin B	[23,24]
	Ciprofloxacin	[25,26]
	Cisplatin	[27]
	Docetaxel	[28]
	Doxorubicin	[29,30]
	Paclitaxel	[31,32]
	Rifampicin	[33]
	r-hGH (recombinant human growth hormone)	[34]
polylactide (PLA)	Camptothecin	[35,36]
	Doxorubicin	[37,38]
	5-fluorouracil	[39]
poly(ε-caprolactone) (PCL)	Amphotericin B	[40]
	Ciprofloxacin	[41]
	Citropin	[42]
	Vancomycin	[43]

## 5. Ring Opening Polymerization of Cyclic Esters 

**Scheme 1:**
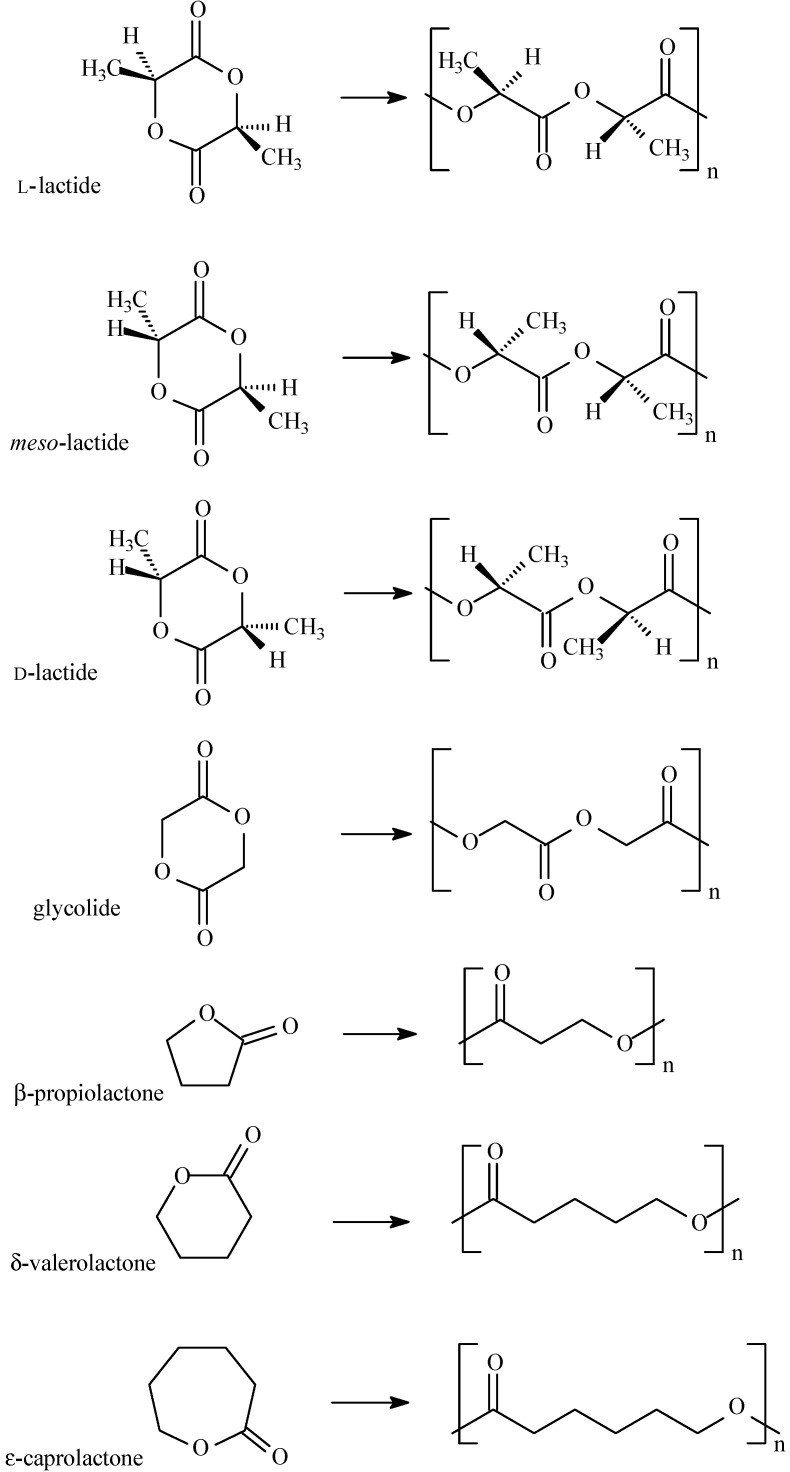
The synthesis of biodegradable polyesters used in DDS technology.

There are currently two main methods for the synthesis of aliphatic polyesters, namely polycondensation and ring opening polymerization (ROP) of cyclic monomers. ROP is the most common method for synthesizing aliphatic polyesters with tailored properties and controlled architecture with desired end groups (which is very important in biomedical applications). The ROP process gives polyesters with high molecular weight, high purity and low polydispersity compared to the polycondensation process, so it is the preferred route in controlled release technology. This method also avoids formation of by-product such as water, the presence of which can cause a shift of the thermodynamic equilibrium of the reaction towards hydrolysis. Several possible combinations of type of monomer, temperature, time, initiators and catalysts have been evaluated to achieve the desired polymer architecture and properties. The ROP of cyclic esters can be performed in the presence of cationic or anionic initiators as well as metal coordinate and enzymatic catalysts [44,45,46]. The scheme of preparation of biodegradable or bioresorbable polyesters used in technology of DDS is shown in Scheme 1.

## 6. Biocatalytic Synthesis of Polymers for Biomedical Applications 

A large number of enzymes catalyze the synthesis of natural polymers such as polysaccharides, proteins or polyesters in living systems [47]. Enzymes can be highly chemoselective, enantioselective and regioselective. They allow for synthesis of macromolecules with well-defined architectures (end-functional polymers). 

Enzyme catalysis has provided a new synthetic strategy for a variety of useful biomaterials, most of which would to be difficult to produce using conventional chemical catalysts [48,49,50,51,52,53,54]. For example, tin(II) 2-ethylhexanoate Sn(Oct)_2_, probably the most often used catalyst in the polymerization of cyclic esters, has been approved by FDA as a food additive. However, it is difficult to completely remove residual organo-tin catalyst, which leads to toxicity problems for biomedical applications [55].

The particular benefits offered by enzymes are high specificity, and high acceleration rate of reactions under mild conditions of pressure, temperature and pH, which makes enzymatic reactions very energy efficient [56].

Enzymes do not require any co-catalyst and they are recyclable materials derived from renewable sources. Moreover, unlike the conventional anionic initiators, large size lactones (macrolides) can be easily polymerized with a high efficiency *via* enzymatic routes [57,58]. The catalytic activity of enzymes in e-ROP process of cyclic esters has been examined.

It has been found that lipases (triacylglycerol acylhydrolase, EC 3.1.1.3) are the most effective enzymes for the synthesis of polyesters (Table 2). They are ubiquitous enzymes that have been found in most organisms from the microbial, plant and animal kingdoms. In living cells lipases catalyze an ester bond-cleavage in water. In low water environments or non-aqueous systems lipases induce the reverse reaction of hydrolysis, leading to polymer production [59,60,61,62,63].

The most important advantage of lipases are their stability under varying environmental conditions (*i.e.*, high temperature, pH). The majority of the industrial enzymes are usually obtained from microorganisms that produce a wide variety of extracellular lipases. Most lipases of microbial origin exhibit their maximum activity in the pH range 7 to 9 and the temperature range from 30 °C to 40 °C. There are also lipases (e.g., lipase from *Thermomyces lanuginosus*) which show up to 25% of their activity at a temperature of 100 °C. Lipases derived from *Rhizomucor miehei*, *Thermomyces lanuginosus* and produced by the yeast *Candida antarctica* (CA) due to their high activity and stability at temperatures around 80 °C are commonly used as biocatalysts on a laboratory and industrial scale [64].

Manufacturers offer lipolytic enzymes both as lyophilized powders and immobilized forms or poly(ethylene glycol)-modified enzymes. Enzyme immobilization on hydrophobic media such as a macroporous acrylic resin, polystyrene or porous silica can significantly increase their activity and thermal stability compared to the free enzyme. Immobilization techniques allows also one to recover the enzymes from the reaction mixtures, providing a continuous process and minimizing the loss of catalyst [65,66,67,68]. However, it is important to take into account the nature of solid state that may affect the suitability of the reaction media [69].

**Table 2:** Origin of lipases commonly used in the e-ROP of cyclic esters.

Origin of Lipases	Ref.
*Aspergillus niger*	[70]
*Candida antarctica*	[45,71,72,73,74,75,76,77,78]
*Candida cylindracea*	[45,70,78]
*Candida rugosa*	[70]
*Penicillium roqueforti*	[70]
Porcine pancreatic	[45,70,78,79,80,81,82]
*Pseudomonas cepacia*	[45,70,76,78]
*Pseudomonas fluorescens*	[45,70,76,78]
*Yarrowia lipolytica*	[83]

### 6.1. Influence of the Reaction Media on Activity of Biocatalysts 

A number of researchers have developed e-ROP of cyclic monomers in organic solvents due to their many advantages: (1) shifting of the thermodynamic equilibrium toward polyester synthesis; (2) increasing the solubility of non-polar products or substrates; (3) simple removal of solvent and biocatalyst; (4) prevention of microbial contamination as a result of the decreased water activity of the environment [84,85,86,87,88]. 

However, small amounts of water bound to the enzyme are required to maintain the catalytic activity of the enzyme [84,89]. Access to the active site may be shielded by a mobile, hydrophobic lid, whose position determines the enzyme conformation. The presence of a minimum amount of water assists the reorientation of the catalyst structure at an interface [90]. In non-aqueous systems enzymes can fold into their native structure provided that the essential water layer around them is not stripped off [85]. Hydrophobic, organic solvents leave the hydration shell of the protein intact due to the small redistribution of water molecules. The amount of water required to maintain the native conformation of biocatalyst is mainly dependent on the nature of the catalyst and the type of environment.

Toluene and heptane are organic solvents commonly used during the synthesis of polyesters. They are known as hazardous, toxic and flammable media. Due to the toxicity of the vapors of these typical solvents, recent literature reports suggest the possibility of using thermal and chemically stable ‘green solvents’- ionic liquids (ILs) as the reaction media. ILs have thus emerged as promising alternative green solvents due to their favorable properties including: (1) negligible vapor pressure; (2) low flammability; (3) wide liquid range; (4) ability to dissolve polar and non-polar, organic, inorganic, organometallic and polymeric compounds; (5) ability to controlled miscibility with many organic solvents and (6) high reusability [91,92,93,94,95,96,97]. Moreover, the use of ILs does not require special equipment and is very energy efficient, which makes this methodology environmentally friendly.

### 6.2. Ionic Liquids

ILs are organic salts with melting points below 100 °C. They are also called ‘room-temperature ionic liquids’, because they remain liquid in the ambient temperature. ILs remain liquid over a wide temperature range from their melting point of approx. −80 °C to their decomposition temperature, which often exceeds 300 °C [91,97,98,99]. 

Unlike traditional solvents, which can be described as molecular liquids, ILs are composed entirely of ions: large, asymmetrical, organic and heterocyclic cation (*i.e.*, tetraalkylammonium, 1,3-dialkylimidazolium; 1,1-dialkylpyrrolidinium, 1,4-dialkylpyridinium, n-alkylpyridinium) and small, inorganic, organic or complexes anions (*i.e.*, tetrafluoroborate, hexafluorophosphate, bis[(trifluoromethyl)sulfonyl]imide, trifluoromethyl acetate, trifluoromethyl sulfate, chloride) [91,94].

Ionic liquids are called ‘tailored solvents’ or ‘designer solvents’ because their physical and chemical properties (e.g., viscosity, density, miscibility with other solvents, melting point) depend on their structure and can be fine-tuned by changing the anion and the cation [100]. The number of possible anion-cation combinations amounts 10^18^ [101]. This allows one to choose appropriate ILs for specific reaction conditions. Given the well-known fact that in some cases enzymes are much more stable in organic solvents than in aqueous solution, it is expected that ILs should have the same effect [89,94,100].

In recent years, ILs have increasingly attracted attention as solvents for biocatalytic polyester synthesis. It has been found that in some cases these “green solvents” improve the solubility of substrates and (or) products and also provide better enzyme stability, activity and selectivity. Moreover, ILs prevent the thermal deactivation of enzymes resulting in the formation of high molecular weight polymers with desired architecture [93]. Enzymatic synthesis of polymers in bulk or in typical organic solvents (like toluene) requires using even 10 times more enzyme in comparison to e-ROP in ILs [74,77,102]. For these reasons, ILs offer new possibilities for the solvent engineering in e-ROP. Those based on 1-alkyl-3-methylimidazolium cations *i.e.*, 1-butyl-3-methylimidazolium tetrafluoroborate, 1-butyl-3-methylimidazolium bis[(trifluoromethyl)-sulfonyl]imide and 1-butyl-3-methylimidazolium hexafluorophosphate are the most commonly used ILs for enzymatic polyester syntheses (Table 3).

**Table 3:** Ionic liquids discussed in this review.

ILs	Abbreviation
1-Butyl-3-methylimidazolium tetrafluoroborate	[bmim][BF_4_]
1-Butyl-3-methylimidazolium bis((trifluoromethyl)-sulfonyl)imide	[bmim][NTf_2_]
1-Butyl-3-methylimidazolium hexafluorophosphate	[bmim][PF_6_]
1-Butyl-3-methylimidazolium trifluoromethanesulfonate	[bmim][OTf]
1-Ethyl-3-methylimidazolium tetrafluoroborate	[emim][BF_4_]
1-Hexyl-3-methylimidazolium hexafluorophosphate	[hmim][PF_6_]
1,4-bis(3-Hexylimidazolium-1-yl)butane bishexafluorophosphate	[C_4_(C_6_Im)_2_][PF_6_]
1-Dodecyl-3-methylimidazolium bis((trifluoromethyl)-sulfonyl)imide	[C_12_MIm][NTf_2_]
1-Dodecyl-3-methylimidazolium hexafluorophosphate	[C_12_MIm][PF_6_]

#### 6.2.1. Enzyme Stability in Ionic Liquids

Enzyme activity and stability can be improved by adjusting the ILs’ properties. High stability and activity of enzymes in ILs are compared with a specific chemical architecture of ionic liquids. ILs are formed by an extensive network of cations and anions linked by hydrogen bonds. For this reason, ILs are named “hydrogen bond polymeric supramolecules” [103]. The inclusion of other molecules in that network allows the existence of both polar and non-polar regions in the IL structure [104]. This arrangement can provide high protection for a biocatalyst. Lozano *et al.* suggest that ILs should be considered as both reaction media and immobilization supports [105]. Moreover, the specific architecture of ILs allows isolation of polymers by extraction using conventional solvents after the completion of a process. Enzymes remain trapped in ILs, which allows one to recover and reuse them for continuous processes, resulting in further process cost reductions [94,100]. However, not all ILs are suitable for biocatalysis. The activity, specificity and stability of the enzyme in ILs depends on the solvent characteristics such as: polarity, hydrophobicity, viscosity, hydrogen-bond capacity, solvent miscibility and impurities [94,100].

#### 6.2.2. Polarity of Ionic Liquids

**Figure 2:**
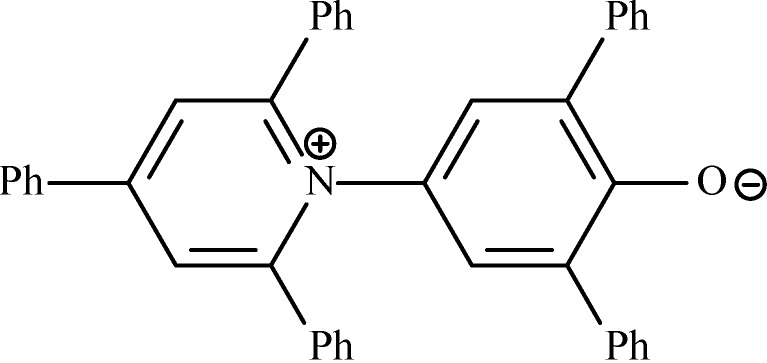
2,6-Diphenyl-4-(2,4,6-triphenyl-1-pyridinio)phenolate (Reichardt’s dye).

Enzyme activity strongly depends on the environment’s polarity. The solvent polarity is usually determined by an empirical method using the solvatochromic dye Nile Red or Reichardt’s dye (Figure 2). The most commonly used method is Reichardt scale (E_T_), wherein the polarity is determined based on the shift of the charge-transfer absorption band of a Reichardt’s betaine, zwitterions, exhibiting negative solvatochromic properties. The shift is due to the hydrogen bonding between the solvent and the phenoxide oxygen atom present in Reichardt’s dye [94,106].

On the normalized polarity scale (E_T_^N^) tetramethylsilane has a polarity of 0.0 and water has a polarity of 1.0. The polarity values of commonly known ILs were found to being close to typical hydrophilic organic solvent *i.e.*, lower alcohols (e.g., methanol, ethanol, 1-butanol) and formamide and varies in a relatively narrow range of 0.6–0.7 [107,108].

The polarity of the imidazolium-based ILs is usually determined by: (1) electrostatic interactions between the anion and cation; (2) the presence of hydrogen bonds between the hydrogen atom of the imidazolium ring and oxygen or halogen atoms in anion; (3) the size of the cation and anion; (4) π–π interactions in aromatic ring. There is a correlation between the decrease of both the alkyl chain length and anion size resulting in increasing polarity [109]. For the 1,3-dialkylimidazolium salts the polarity decreases with increasing anion size ([BF_4_] > [PF_6_] > [NTf_2_]), hence a reduction in the effective anion charge density [107].

High polarity values suggest that ILs should exhibit strong hydrogen bonding forces [92]. In general, highly polar media can strip the water from an enzyme due to the fact they can hold water in solution and decrease the activity and selectivity properties of a biocatalyst [62,65,92,110,111,112].

The hydrogen bonding could be the key to understanding the interactions between enzymes and ILs. IL anions can form strong hydrogen bonds that maintain the structural integrity of the enzyme’s α-helices and ß-sheets, but also it could cause the catalyst’s deactivation. Cations with acidic properties *i.e.*, pyridinium, maintain the enzyme activity [113]. More basic anions may interfere with the enzyme’s internal hydrogen bonds [102].

As mentioned earlier, ILs show polarity values comparable to typical organic solvents, but surprisingly they do not inactivate enzymes. Decreased water activity of the enzyme micro-environment as a result of the high polarity of ILs may improve the activity and selectivity of enzymes [114,115]. Lozano *et al.* reported that an increase in the polarity of the imidazolium-based ILs enhanced both synthetic activity and selectivity of free *Candida antarctica* lipase B (CALB) at 2% (v/v) water content in comparison with two typical organic solvents (hexane and 1-butanol) [105]. However, small differences between the polarity values of ILs suggest that there may be no correlation between lipase activity and the normalized polarity scale [116].

#### 6.2.3. Hydrophobicity/Hydrophilicity

Despite their high polarity, most of the ILs commonly used in biocatalytic processes are hydrophobic as a result of the presence of alkyl substituents in the cation molecules. For e.g., [hmim][PF_6_] is more hydrophobic (590 ppm of water content obtained after 4 h drying at 70 °C) than [bmim][PF_6_] (472 ppm under the same conditions) [117].

They are miscible with polar solvents, *i.e.*, dichloromethane, tetrahydrofuran, but not miscible with *i.e.*, hexane. The miscibility of ILs with water varies widely and unpredictably. Although three ILs based on the 1-butyl-3-methylimidaziolium cation: [bmim][PF_6_], [bmim][NTf_2_] and [bmim][BF_4_] have similar polarity (Table 4), the first two ILs are only slightly soluble in water (0.13 and 1.40% v/v respectively), whereas the latter is completely miscible with water [100]. It is well known that hydrophilic ILs with nucleophilic anions are an ideal medium for polar substrates. However, in biocatalysts, hydrophilic solvents can form covalent interactions (hydrogen bonding) with the active center of the enzymes and cause a decrease in their activity. Therefore, most hydrophilic ILs act as enzyme deactivating agents, with few exceptions, *i.e.*, [bmim][BF_4_], [emim][BF_4_], [bmim][OTf] [69,113,116].

**Table 4:** Physicochemical properties of ILs commonly used in biocatalysis.

ILs	Polarity E_T_^N^ (RT)	*M_w_* (g·mol^−1^)	Melting Point (°C)	Viscosity in 20 °C (cP)	Density (g·cm^−3^)	Conductivity (S·m^−1^)	Water Solubility (WS) (%w/v)
[bmim][BF_4_]	0.680	226	−82	233	1.17	0.17	100
[bmim[Tf_2_N]	0.645	419	−4	52	1.43	0.39	1.40
[bmim][PF_6_]	0.676	284	−8	312	1.362	0.14	0.13

Kim *et al.* reported enhanced enantioselectivity of CALB in ILs compared to a typical organic solvent (toluene). Moreover they reported that lipases show higher enantioselectivity in the hydrophobic IL [bmim][PF_6_] compared to the hydrophilic IL [emim][BF_4_] [118]. Furthermore, hydrophobic ILs have stabilizing effect on hydrolases in non-aqueous environments due to the fact ILs maintain the native conformation of enzymes [69].

Because of the high hydrophobicity of ILs, most lipases do not dissolve in IL environments, but remain in suspension while maintaining high catalytic activity [119]. However, van Rantwijk *et al.* found, that the negative, structural changes of the active site of enzymes, which take place during dissolution in ILs, are reversible [95].

#### 6.2.4. Viscosity

As is shown in Table 4, ILs are highly viscous solvents with viscosity that vary in a relatively narrow range of 35–500 cP, compared with the viscosity of toluene at 25 °C which is 0.6 cP, and that of water, 0.9 cP [94,99,108]. The high viscosity values of ILs are the result of strong intermolecular forces (charge-charge interactions, van der Waals forces) [100]. Increased viscosity is a result of the elongation of the cation’s alkyl chain [94]. High viscosity may slow down the conformational changes of biocatalysts, allowing them to maintain their native structures [116,120,121]. Due to the small differences in the polarity of ILs, some reports suggest that solvent viscosity has significant effect on reaction rates and yields [108]. High viscosity values could affect the reaction rate by limiting mass transfer. The addition of organic co-solvents or water can decrease the ILs’ viscosity values. Increasing the temperature has the same effect [93,116,122].

#### 6.2.5. Effect of Impurities

An important factor affecting the enzyme activity and polymer yield is the solvent’s purity. Contaminants that may be present in ILs are halide anions (*i.e.*, chloride ions), organic compounds and water. Chloride ions may constitute contamination due to incomplete ion exchange during the ILs’ synthesis from their chloride precursors. Chloride content below 0.1% w/v does not affect lipase activity, but higher concentrations can cause decreased polymer yields and enzyme deactivation [96]. Madeira Lau *et al.* reported that reduction of the water content in ILs also gives better polymer yields [91].

## 7. Ionic Liquids as a Medium for the Enzymatic Ring-Opening Polymerization of Cyclic Esters

Uyama and Kobayashi were the first to report the e-ROP of ε-caprolactone (CL) in the presence of two ILs based on 1,3-dialkylimidazolium salts: [bmim][PF_6_] and [bmim][BF_4_] (Table 5). Reactions was carried out in the presence of *Candida antarctica* (CA) lipase at 60 °C in 24–168 h. They obtained polyesters with a number average molecular weight (*M_n_*) in the range from 300 to 4200 g·mol^−1^. Polymers with the higher *M_n_* were obtained using as solvent [bmim][BF_4_] after a reaction time of 168 h. The authors reported also high polydysperisity (*PDI*) values for the polymers ranging from 2.7 to 7.3 [123].

Marcilla *et al.* also investigated the e-ROP of CL in ILs with three different anions: [bmim][X] (X = BF_4_, PF_6_, NTf_2_) at 60 °C for a reaction time of 24 h, using immobilized lipase B from *Candida antarctica* (Novozym 435) as a catalyst. They obtained oligomers with *M_n_* in the range from 10,500 to 12,700 g·mol^−1^ and *PDI* values range from 1.7 to 2.1 after precipitation [124].

Although the abovementioned reactions were carried out under the same conditions, there were significant differences in the polymers’ *M_n_* values as a result of the differences in: (1) amounts of monomer and catalyst used; (2) forms of the enzymes (free and immobilized lipases); (3) purity of ILs; (4) the presence of the precipitation process that provided a higher polymer *M_n_* and lower *PDI*.

**Table 5:** Ring opening enzymatic polymerization and copolymerization of cyclic esters in ionic liquids.

Monomer	ILs	Purity of ILs (%)	Enzyme	Temp. (°C)	Time (h)	M_n_ (Da)	*PDI*	Y/C (%)	Ref.
	[bmim][BF_4_]	-	CA	60	168	4200	2.7	97^C^	[123]
	[bmim][BF_4_]	≥99	CA	60	24	12,700	1.8	35^Y^	[124]
	[bmim][BF_4_]	-	YLL	100	16	1758	1.7	-	[113]
	[bmim][BF_4_]	-	YLL	150	6	3092	2.5	-	[113]
	[bmim][PF_6_]	-	CA	60	72	540	4.2	62^C^	[123]
	[bmim][PF_6_]	≥99	CA	60	24	12,200	1.7	30^Y^	[124]
CL	[bmim][NTf_2_]	≥99	CA	60	24	10,500	2.1	44^Y^	[124]
	[bmim][NTf_2_]	≥97	CA	90	24	8100	-	85^Y^	[102]
	[C_4_(C_6_Im)_2_][PF_6_]	-	CA	90	48	26,200	-	62^Y^	[125]
	[C_12_MIm][PF_6_]	-	CA	90	48	11,700	-	37^Y^	[125]
	[C_12_MIm][NTf_2_]	-	CA/ILs	60	48	35,600	-	62^Y^	[125]
	[C_12_MIm][NTf_2_]	-	CA	60	48	20,300	-	54^Y^	[125]
LLA	[bmim][BF_4_]	≥99	CA	110	24	54,600	1.25	24.3^Y^96.2^C^	[126]
	[bmim][PF_6_]	≥99	CA	65	264	581	1.2	29.5^Y^	[127]
	[bmim][PF_6_]	-	CA	90	120	19,600	1.2	-	[128]
	[bmim][PF_6_]	≥99	CA	120	24	3900	1.19	0.1^Y^90.4^C^	[126]
	[hmim][PF_6_]	≥97	CA	90	168	37,800	1.3	63.2^Y^	[129]
	[hmim][PF_6_]	≥97	CA	65	120	1700	1.3	16.5^Y^	[129]
	[bmim][NTf_2_]	≥99	CA	120	24	50,100	1.42	10.5^Y^	[126]
DO	[bmim][PF_6_]	≥99	CA/ILs	70	18	182,100	-	-	[130]
	[bmim][PF_6_]	≥99	CA	70	24	27,700	-	-	[130]
GA	[bmim][PF_6_]	≥99	CA	65	96	-	-	-	[127]
LLA:GA (1:3)	[hmim][PF_6_]	≥97	CA	65	120	3500	1.3	40.2^Y^	[129]
	[bmim][PF_6_]	≥99	CA	65	96	2400	1.1	36.7^Y^	[127]
LLA:GA (3:1)	[bmim][PF_6_]	≥99	CA	90	144	18,500	1.1	-	[127]

**CL**: ε-caprolactone; **LLA**: l-lactide; **DO**: (1,4-dioxane)-2-one; **GA**: glycolide; **LLA:GA**: molar ratio of l-lactide to glycolide; **CA**: lipase from *Candida antarctica*; **YLL**: lipase from *Yarrowia lipolytica*; **CA/ILs**: lipase from *Candida antarctica* coated with ionic liquids; **PDI**: polydispersity index; **Y**: yield; **C**: conversion.

### 7.1. Effect of Monomer and Polymer Solubility in ILs

Low solubility of LA in commonly used organic solvents restrict enzymatic polymerization to bulk reactions. The polar and hydrophilic environment of melted LA results in lipase deactivation (especially at high temperature), limiting the polymer propagation. Yoshizawa-Fujita *et al.* showed that at relatively high temperature (110 °C) in IL media ([bmim][NTf_2_] and [bmim][BF_4_]) one can obtain high *M_n_* poly-l-lactide (PLLA) (54,600 g·mol^−1^) compared to toluene and bulk (42,200 g·mol^−1^ and 15,600 g·mol^−1^ respectively) [126].

In the case of CL, despite the high solubility of the monomer in two ILs based on 1,3-dialkylimidazolium cations: [bmim][BF_4_] and [bmim][PF_6_], the polymer product miscibility is strongly dependent on the nature of the IL anion. It is very important to choose appropriate ILs for polymer synthesis. Poor solubility of the polymer in the ILs could lead to a low molecular weight of products due to the premature precipitation of the polymer from the reaction mixture. On the other hand, better solubility of the polymer in ILs most likely leads to lower polymer yields due to difficulty of polymer extraction from the solvent [102]. Compared to the typical organic solvent, toluene, Marcilla *et al*. showed the lowest *M_n_* of PCL for the reaction conducted at 60 °C in an IL medium due to the differences in polymer’s solubility [124]. Marcilla *et al.* suggested that when polymer is insoluble in ILs it is diffusing through the layers of a heterogeneous reaction mixture and this provides a limited monomer conversion degree. The polymerization process carried out under these conditions is, therefore, closer to bulk polymerization than the solution type [124]. Moreover, the heterogeneous system facilitates the polymer extraction from the reaction mixture after the completion of the process [113]. Yoshizawa-Fujita *et al.* and Marcilla *et al.* reported that the lowest polymer yield for e-ROP ocurred in [bmim][PF_6_] due to the lowest solubility of the obtained polymers in the IL. The relative solubility values of PLLA in different ILs are in the following order: [bmim][NTf_2_] > [bmim][BF_4_] > [bmim][PF_6_] [124,126].

### 7.2. Effect of Temperature

Hydrophobic ILs provide better stability of an enzyme compared to hydrophilic solvents, specially at high temperatures. Gorke *et al.* reported the influence of temperature on the e-ROP of CL using as catalyst free CALB in [bmim][NTf_2_]. The process was carried out in various temperatures: 25, 60, 90, 100 and 110 °C for 24 h. At 25 and 60 °C, *M_n_* continued to increase till it reached a maximum at 90 °C. Decreased *M_n_* and polymer yield at higher temperatures could be a result of rapid inactivation of the enzyme under those conditions [102].

Yoshizawa-Fujita *et al.* reported the same effect in the lipase-catalyzed synthesis of PLLA in ILs based on 1-butyl-3methylimidazolioum cations: [bmim][NTf_2_], [bmim][BF_4_], [bmim][PF_6_]. However, they reported the highest *M_n_* of PLLA in the 110 to 120 °C temperature range, and further temperature increases caused the deactivation of the enzyme [126]. 

Barerra-Riverra *et al.* reported decreased monomer (CL) conversion in the presence of enzyme (*Yarrowia lipolytica* lipase, YLL) due to the increased temperature in range of 60 to 90 °C for hydrophilic ILs. This is probably a result of the deactivation and denaturation of the enzyme at relatively high temperatures. Thus, hydrophobic ILs provide better temperature stability of enzymes at high temperatures compared to hydrophilic ILs [113].

As mentioned earlier, ILs are solvents with relatively high viscosity values of up to 500 cP although most of them display Newtonian behavior over a wide temperature range. High viscosity retards mass transfer but increasing temperature can decrease this value. Therefore it is important to describe the solvent viscosity at the polymerization temperature [129]. Chanfreau *et al.* reported relatively low viscosity values for [hmim][PF_6_] at 90 °C (<30 cP) compared to viscosity at 1 °C (12,000 cP) which was not expected to be a limiting factor during polymerization in that case [129].

Lipase-mediated polymerization in ILs is a promising method for the synthesis of functional polyesters: PCL, PLA and copolymers of LA and glycolide (GA). However, for LA and GA under relatively high temperature polymer synthesis without catalyst was observed. This probably results from a cationic mechanism induced by traces of hydroxyacid from the monomer. The effect is strongly dependent on the temperature, type of monomer and solvent. For GA polymerization it takes place in [bmim][PF_6_] even at 65 °C, whereas for LA polymerization the same effect was observed at 90 °C in [hmim][PF_6_] and none was observed at 65 °C [127,131].

### 7.3. Effect of Enzyme Preparations

The water activity in IL systems are the key factors for evaluation of biocatalytic reactions in organic solvents*.* Increasing the enzyme concentration may lead to hydrolysis as a result of a higher water content in the reaction environment. It has been shown that reduction of water content as a result of the pre-drying of enzymes could improve the catalytic activation of lipases. Gorke *et al.* carried out the polymerization of CL, β-propiolactone and δ-valerolactone using CALB as catalyst and [bmim][NTf_2_] as a reaction medium during 24 h at 60 °C. Pre-treatment of the enzyme allowed them to obtain higher *M_n_* polymers compared to catalyst without pre-drying [102].

As mentioned earlier, ILs can be used both as solvent and as in the pre-treatment of enzyme process. Coating of an enzyme by an IL layer increases the activity, stability and enantioselectivity of the biocatalyst [130,132]. Moreover, coating enzymes by IL layers helps to reduce the IL cost during the synthesis. The coating of the enzyme by an IL layer is based on absorption of ILs on the polymer surface without using chemical bonds. Dong *et al.* carried out the e-ROP of (1,4-dioxane)-2-one (DO) in the presence of immobilized CALB (Novozym 435) coated with [bmim][PF_6_], to obtain a polymer having a *M_n_* of 182,000 g·mol^−1^ [130]. Wu *et al*. showed a similar relationship using Novozym 435 coated by [C_12_Mim][NTf_2_] to obtain PCL having a *M_n_* of approx. 35,600 g·mol^−1^. Use at the same as the above conditions of ILs acting as reaction medium leads to the preparation of polymer with a *M_n_* value of 20,300 g·mol^−1^ [132]. Enzyme pre-incubated in ILs before polymerization can improve the catalytic activity of CALB and this procedure allows one to obtain higher polymer *M_n_* values [130].

### 7.4. Structure of Polyesters Obtained in ILs 

Well-defined macromolecules with appropriate molecular weight, multifunctional terminal groups, narrow polydispersity and steroregularity are receiving increasing attention in biomaterial applications, especially in DDS systems. As mentioned earlier, polymers with suitable structure can achieve more predictability and reproducibility release of APIs.

As shown by Marcilla *et al.* the *PDI* of polyesters synthesized in [bmim][BF_4_] and [bmim][PF_6_] are lower (*PDI* = 1.7–1.8) compared to those obtained in toluene (*PDI* = 2.3) [124]. Besides the formation of linear polymers, e-ROP catalyzed by lipases in organic solvents (*i.e.*, toluene, heptanes, ILs) favored the formation of macrocycles as a result of inter- and intra-molecular transestrification, end-to-end condensation and backbiting (intermolecular termination) [130,133].

The structure of the obtained polymers depends on the temperature, time, monomer concentration and the nature of the ILs. At low monomer concentrations, mainly linear polymers are obtained in ILs. Prolonged reaction times at 60 °C lead to the formation of low molecular weight products as a result of cyclic species formation [113]. However, the spectra of the polymers obtained in ILs display a lower ratio of rings to linear polymers in comparison to those obtained in heptane and toluene [113,124].

Hyperbranched polyesters (HBPs) are very interesting biomaterials. These polymers are considered to be very attractive for DDS technology. HBPs have been the first obtained in the e-ROP of CL by using CALB with bis(hydroxymethyl)butyric acid (BHB) as AB_2_ core in toluene/dioxane mixtures. The degree of branching (DB) of these polymers ranged from 0.03 to 0.33 [134]. López-Luna and co-workers reported the CALB-mediated synthesis of HBPs using BHB in liquid 1,1,1,2-tetrafluoroethane media. In this case the DB was rather low (0.02–0.09) [135]. Recently, imidazolium-based ILs have also been shown to be adequate media which enable sustaining enzyme activities. Moreover, they are a good solvent for LA. The obtained PLA was characterized with rather high molecular weights. In [128] the method of synthesis of hyperbranched PLA in the presence of [bmim][PF_6_] has been described. BHB has been used as the AB_2_ core co-monomer and immobilized CALB as biocatalyst. The DB of obtained HBPs has been controlled by the reaction conditions (the maximum value was 0.21) [128].

### 7.5. Kinetics of Polyester Enzymatic Synthesis in ILs

The effect of IL type on the kinetics of e-ROP of cyclic esters has been discussed by many authors. The results indicate that high monomer conversion was possible when the process was carried out for long periods of time at relatively low temperatures. For example Uyama and Kobayashi showed an increased monomer conversion of CL at 60 °C in [bmim][BF_4_] from 23% to 97% for 24 to 168 h. A similar effect was reported for e-ROP carried out in [bmim][PF_6_] [123].

However, general conclusions about the effect of temperature on the kinetics of e-ROP of polyesters cannot be drawn. High temperatures can cause inactivation of the biocatalyst, although for PLLA synthesis in [bmim][BF_4_] high LLA conversion from 67.6% to 100% was reported as a result of increasing the temperature from 90 to 120 °C [126].

Dong *et al*. reported monomer conversion of DO in [bmim][PF_6_] in different temperature ranges from 40 to 80 °C using CALB as biocatalyst. The result was an increased conversion value from 30% to 60% at the temperature range from 40 to 70 °C. At higher temperature the conversion value decreased as a result of enzyme denaturation processes. Prolonged polymerization time (from 10 to 30 h) at 70 °C caused an increased conversion value from 54% to 61% in the case of ILs used as a solvent and from 57% to 62% in the case of CALB coated by an IL layer [130].

## 8. Conclusions 

Biodegradable or bioresorbable polyesters are very important biomaterials expected to make essential contributions to the development of pharmaceutical science in the future. The enzyme-catalyzed ring opening polymerization of lactones or lactides seems to be a promising method of biomedical polyester synthesis. ILs offer new possibilities for solvent engineering in e-ROP of cyclic esters. Many authors believe that e-ROP of cyclic esters in the presence of ILs will be an alternative method for the preparation of functional, stereo- and regioregular biomedical polymers. In DDS technology it is very important to use structurally well-defined matrices. The kinetics of active substance release depend not only on the *M_n_* of the polymer, but polydispersity index, appropriate degree of crystallinity and stereoregularity of the polymer are equally important factors in pharmaceutical fields. The stabilization and chemo- regio- and stereoselectivity of enzymes in ILs are very important aspects of biomedical polymer synthesis. Moreover, some of the polyesters synthesized by e-ROP (in the presence of ILs) have been shown to have higher molecular weight and higher monomer conversion compared to typical organic solvents. However, the total control of the *M_n_* of polymers in e-ROP process is still limited. The use of ILs in the e-ROP of cyclic monomers definitely needs further exploration.
